# Axial Compression Property Test of GFRP Tube-Confined Coal Gangue Steel Fiber Short Concrete Column

**DOI:** 10.3390/polym14214528

**Published:** 2022-10-26

**Authors:** Shengyong Xia, Haiqing Liu, Guosheng Fu, Jinyang Zhang, Ming Lei, Zimu Chen

**Affiliations:** 1 School of Civil Engineering, Liaoning Technical University, Fuxin 123000, China; 2 China Construction Fifth Engineering Division Corp., Ltd., Changsha 410004, China

**Keywords:** GFRP tube, steel fiber, coal gangue concrete, axial compression test

## Abstract

In order to study the axial compression property of a GFRP (glass fiber-reinforced polymer) tube-confined coal gangue steel fiber short concrete column, a test was carried out. The whole process of deformation and failure of the specimen under axial compression load was observed, and the whole process of the stress–strain curve of the specimen was obtained. The results show that the thickness of the GFRP tube has the most significant effect on the mechanical properties. The thickness of the 7 mm tube is 4.3 times the axial ultimate stress and 21.5 times the ultimate strain of the unconstrained short column. Under a certain volume fraction, the ultimate axial strain of the wave fiber is 10.1% higher than that of the hook fiber short column, and the ductility coefficient is 9.6% higher. The fiber volume fraction significantly increases the strain of the short column, and the 3% fiber content is 50.1% higher than that of the non-fiber short column. Finally, three classical strength models of confined concrete were selected for comparative calculation, and a new stress correction model was proposed.

## 1. Introduction

With the vigorous development of the construction industry, the demand for concrete is gradually increasing. As an essential concrete component, the traditional aggregate resources represented by common stones are decreasing. In addition, mining coal produces a significant amount of coal gangue, which is removed from the ground and left to pile around the mine for an extended period, taking up much space and seriously harming the area’s ecology [1,2,3,4]. Making concrete with coal gangue as aggregate not only solves the problem of traditional aggregate resource scarcity but also improves the current situation of coal gangue pollution of the environment and realizes the secondary use of industrial waste [5]. However, studies have shown that coal gangue has low strength, large porosity, and water absorption compared to ordinary stone [6], which also directly limits the application of coal gangue concrete in engineering.

Lateral restraint can effectively improve the mechanical properties of concrete members. As a traditional concrete constraining method, steel tube confined concrete has good properties in improving both the strength and ductility of concrete. However, as a common external constraint of concrete, a steel tube has certain limitations. When the steel tube reaches yield stress, it cannot provide continuous restraint for the core concrete [7]. In addition, steel tubes are prone to corrosion and poor durability when exposed to saline or a wet environment for a long time [8,9].

Fiber-reinforced polymer (FRP) has the advantages of being lightweight as well as having high strength, stable properties, high mechanical strength, and corrosion resistance [10,11]. It is widely used in the fields of construction engineering, bridge engineering, and underground engineering [12,13,14] to make up for the shortcomings of steel tube-confined concrete in terms of binding force and durability. GFRP tubes provide the binding force and act as permanent formwork [15], so the application prospect is more extensive. Under axial pressure, GFRP tubes provide lateral restraint to put the internal concrete in a three-dimensional stress state, which improves the strength and ductility of concrete to a certain extent [16]. Mingqiao et al. [17] investigated the effect of the GFRP tube-forming process on the bearing capacity of self-compacting concrete by conducting axial compression tests on short columns of GFRP-confined concrete. Yuqiang et al. [18] explored the axial compression mechanical properties of short columns of elliptical GFRP tube-confined concrete with the cross-section ratio and the number of layers of glass fiber cloth as variables. Rodsin et al. [19] strengthened broken brick aggregate concrete square columns with LC-GFRP sheets, observed significant improvements in ultimate compressive stress and corresponding strain, and found a positive correlation with the number of LC-GFRP sheets. Since FRP-confined concrete is passively restrained, the small transverse deformation of concrete cannot give full play to the property of FRP. At the same time, the high brittleness causes temporary softening of the axial force. More FRP materials are needed to provide sufficiently large restraint stiffness to reduce this effect. Steel fibers are an effective way to enhance the deformation property and improve the brittleness of concrete [20], so FRP-confined steel fiber concrete is an excellent composite column. Xie [21] investigated the axial compression property of FRP tube-confined steel fiber high-strength concrete columns and found that the concrete type, fiber shape, and fiber length-to-diameter ratio also affected the property of the specimens but were not too significant. Gholampour [22] studied the axial compression test of steel fiber concrete columns under the action of FRP tube confinement and proposed a finite element model to predict the compressive behavior of FRP-confined steel fiber concrete in circular sections.

In this paper, based on the external confinement GFRP tube to improve the compressive properties of concrete, the coal gangue concrete itself is also improved. Fiber type and fiber content can also improve the mechanical properties of coal gangue concrete. A variety of variables together have an impact on the mechanical properties of coal gangue concrete short columns, and now, there are a few relevant experimental and theoretical studies in this aspect. Through experimental study, GFRP tube thickness contributes the most to the axial compression performance of short columns. The axial compression performance of the wave fiber short column is better than that of the end hook fiber short column. Increasing the fiber volume fraction will improve the mechanical properties when the fiber volume fraction is low. The strength prediction formula of the short concrete column is explored to provide a reference for engineering applications.

## 2. Test Program

### 2.1. Specimens Design

A total of 30 short concrete columns restrained by GFRP tubes were made in 10 groups, three in each group, with the main parameters of the wall thickness of GFRP tubes, fiber type, and volume replacement rate of steel fibers. The column height was 300 mm, and the diameter was 150 mm. The concrete specimens were produced simultaneously. The measured strength of the concrete specimens was 41.6 MPa. To prevent the failure of the end of the specimen, the upper and lower ends of the specimen were winding 1 m long, 5 cm wide CFRP cloth to strengthen the constraints. The surface treatment of the specimens and the adhesion of the fiber cloth were carried out according to the Technical Specification for Carbon Fiber Sheet Reinforced Concrete Structures [23] (CECS146:2003). The loading method was axial compression loading, and the loading rate was 0.2 mm/min until specimen failure. The main parameters are shown in Table 1.

### 2.2. Specimens Production

**Figure 1:**
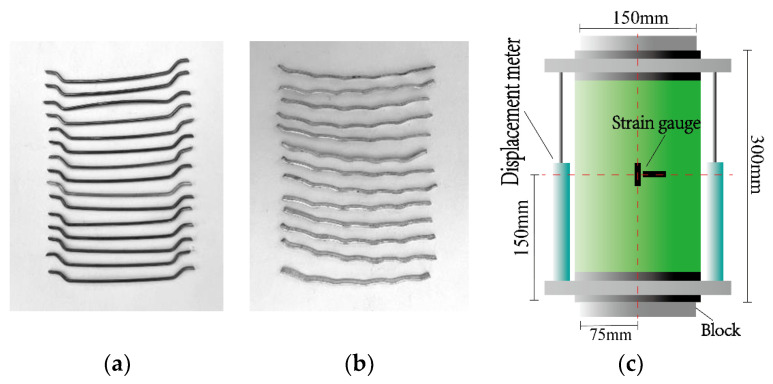
Fiber type and specimen design. (**a**) End hook fiber. (**b**) Wave fiber. (**c**) Specimen design.

**Figure 2:**
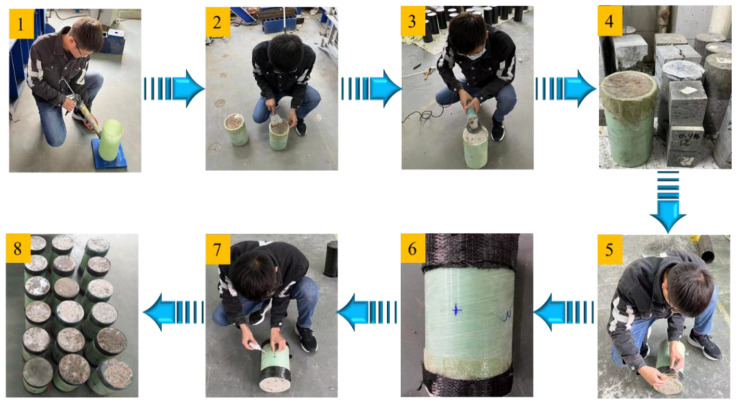
Specific fabrication process of specimen.

The specific production process of the specimen was divided into eight steps. (1) GFRP tube processing: A GFRP tube was cut into 300 mm high short columns. All the tubes were fixed on the padding plate with glass glue. Tubes of the same thickness were placed in a row. (2) Concrete pouring: The test used a HJW-60 single horizontal shaft forced concrete mixer to mix concrete. The steel fiber shape is shown in Figure 1a,b; one was the end hook steel fiber, and another was the wave steel fiber. The mixed concrete was thoroughly vibrated into the GFRP tube, and three concrete cubes (100 mm × 100 mm × 100 mm) were poured. (3) End grinding: After the concrete pouring was completed, the top concrete was ground to the height of the GFRP tube. (4) Standard curing: The upper end was sealed with plastic film, and the cube test block and the cylindrical specimen were cured under the same conditions so that the concrete strength was tested after 28 days. (5) Bonding CFRP cloth: After one week of concrete pouring, the specimen was removed from the padding plate, and the surface of the specimen was wiped clean with clean water. According to the CFRP bonding procedure, 1 m long and 5 cm wide CFRP cloth was pasted on the upper and lower ends of the specimen with matching epoxy resin glue. (6) Label paste position: The middle of the specimen, at 150 mm, was marked with a marker pen, as shown in Figure 1c. (7) Paste strain gauge: To measure the transverse and longitudinal strain gauge, we divided the specimen into two groups with 302 glue symmetrical paste. After pasting, we tested the strain gauge performance one by one with a EM33D type multimeter. (8) To be loaded specimen: We completed the production process, and the specimen were ready to load. The specific preparation process of the specimen is shown in Figure 2

### 2.3. Material Properties and Mechanical Properties

The coal gangue concrete mix proportion is shown in Table 2, and the GFRP tube and CFRP cloth material index are shown in Table 3.

### 2.4. Loading and Measuring Device

**Figure 3:**
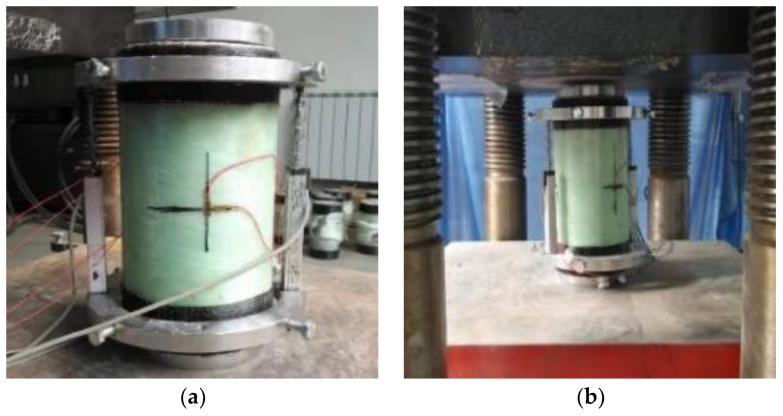
Loading device and specimen arrangement. (**a**) Specimen arrangement. (**b**) Loading device.

As shown in Figure 3, these axial compression test specimens were carried out on the YAW-5000J microcomputer-controlled electro-hydraulic servo compression and shear testing machine. Two 10 cm displacement meters were arranged on both sides of the specimen to test the axial displacement of the deformation zone of the specimen during loading. The specimen’s upper and lower end surfaces were levelled by a grinder. The core concrete was pressed to ensure that the GFRP tube only played a restraining role, and the loading rate was 0.2 mm/min until the specimen failed.

## 3. Test Results and Discussions

### 3.1. Specimens Failure

**Figure 4:**
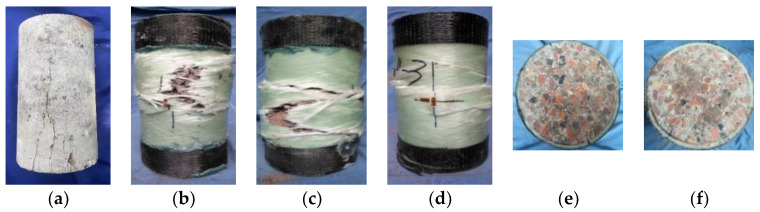
Failure pattern of the specimens. (**a**) T0. (**b**) T3E1. (**c**) T5E1. (**d**) T7W1. (**e**) T5E1 upper surface. (**f**) T7W1 upper surface.

Figure 4 shows the cylindrical specimen’s typical failure mode under the axial compression test. Figure 4a shows that the specimen showed splitting failure for unconstrained specimens. When the load reached the peak, multiple vertical cracks appeared simultaneously, and the crack surface was neat. At the moment of destruction, individual vertical cracks ran through the entire specimen, which was accompanied by a splitting sound. It was a typical splitting failure mode.

For confined specimens, at the initial loading stage, the lateral expansion of concrete was small, the GFRP tube did not play a restraining role, and there was no apparent change outside the specimen. With the further increase in load, the circumferential expansion of the specimen was slight, and some areas outside the GFRP tube began to whiten. When the load was close to the peak load, the sound of minor fracture of the fiber was gradually accelerated, and the GFRP tube fiber was gradually broken. Then, the GFRP tube broke instantly, and the specimen was destroyed. As shown in Figure 4b, the specimens with a wall thickness of 3 mm mainly were tearing failures. The GFRP tube burst from the middle, the fiber and the epoxy resin were broken, the cracks were concentrated in the axial direction, and the cracking range was extensive. The concrete was crushed and flowed out from the tear, and the upper and lower ends of the CFRP cloth were not damaged. As shown in Figure 4c, the specimen with a wall thickness of 5 mm showed a mixture of tearing failure and shear failure. The crack decreased and had a certain angle with the axial direction. As shown in Figure 4e, the concrete and GFRP tube did not appear to be peeling, and the visible concrete and GFRP tube were almost simultaneously damaged. As shown in Figure 4c, the specimen with a wall thickness of 7 mm showed a relatively single shear failure, with a large angle between the cracking and axial directions. Some fibers in the GFRP tube broke, the epoxy resin split along the fiber winding direction, and the upper and lower ends of the CFRP cloth had apparent damage. Observing the upper surface (Figure 4f), the concrete and the GFRP tube were peeled off. The core concrete was crushed before the damage to the GFRP tube occurred.

### 3.2. Analysis of Factors Affecting the Test

**Figure 5:**
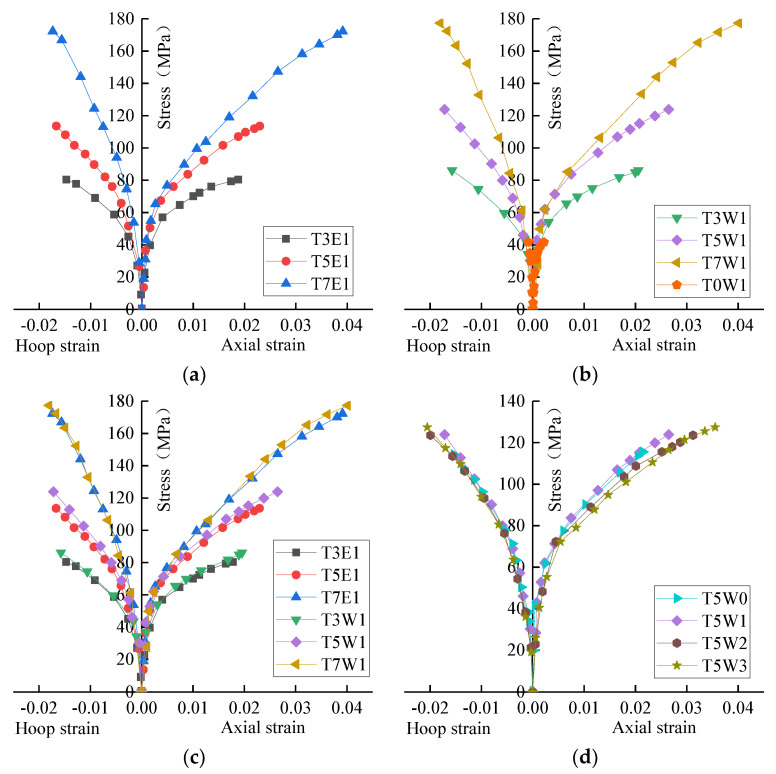
Specimen stress and axial and hoop strain. (**a**) Different tube thickness. (**b**) Different tube thickness. (**c**) Different fiber shapes. (**d**) Different fiber volume fraction.

The stress and hoop and axial strain curves are shown in Figure 5. The specimen was in hoop tension and axial compression during the loading process. The negative value in the figure was the hoop strain, and the positive value was the axial strain. As seen in Figure 5, the compressive strength of the specimen gradually increased with the increase in GFRP tube thickness. The hoop strain gap was small, but the tube thickness change significantly affected the axial strain.

#### 3.2.1. Effect of GFRP Tube Thickness

Figure 5a,b show the axial stress–strain relationship and circumferential stress–strain relationship of GFRP tube thickness on the specimen. It can be seen that the stress–strain curves of all specimens almost show bilinear characteristics, which are smoothly connected by two approximately linear ascending segments and intermediate transition segments. The analysis of Figure 5b showed that the tube thickness of 3 mm, 5 mm and 7 mm was 2.1 times, 3.0 times and 4.3 times the unconstrained specimen in terms of ultimate stress, 9.4 times, 12.1 times and 18.3 times the unconstrained specimen in terms of ultimate axial strain, and 18.8 times, 20.5 times and 21.5 times of the unconstrained specimen in terms of ultimate circumferential strain. With the increase in GFRP tube thickness, the slope of the second stage of the curve increased, and the ultimate stress and strain at the end of the curve increased.

The first stage curves of different GFRP tube thickness specimens almost coincided. In the initial stage, the core concrete of each specimen ignored the lateral expansion, and the GFRP tube had almost no constraint on the core concrete. After entering the transition section, the micro-cracks inside the concrete continued to develop, and the strain growth rate of the GFRP tube accelerated. The strain change in the second stage was mainly affected by the thickness of GFRP. The slower strain growth rate was caused by the GFRP tube with greater restraint stiffness and thickness.

#### 3.2.2. Effect of Steel Fiber Shape

Figure 5c shows the effect of fiber shape on the axial circumferential stress–strain behavior of the specimen. The curve in Figure 5c shows that wave fibers perform better than end-hook fibers in improved compression behavior. A group of specimens with a tube thickness of 3 mm was selected. The wave fiber specimens’ ultimate strength and axial strains were 7.2% and 10.1% higher than the end hook fiber. In the specimen with a tube thickness of 3 mm, the circumferential ultimate strain of the wave fiber specimen increased by 6.7% compared with the end hook fiber. The wave fiber had improved in stress and strain compared to end hook fiber.

In terms of stress–strain behavior, wave fibers had more obvious improvement than end-hook fibers. The geometry of wave fibers had the following advantages over end-hook fibers, resulting in better integrity: the contact area between wave shape and substantial cementitious material increased; the wave shape increased the bonding strength with the cementitious material; regular wave shapes evenly distributed the load transmitted by the cementitious material.

#### 3.2.3. Effect of Fiber Volume Fraction

Figure 5d shows the effect of fiber volume fraction on axial compression. The specimens were made with the same concrete mix ratio. The stress–strain curve showed that the increase in fiber volume fraction led to an increase in the compressive strength and strain of the specimen. Compared with the specimens without fiber incorporation, the ultimate stress of the specimens with fiber volume fractions of 1%, 2% and 3% increased by 7.2%, 7.0% and 10.3%, the ultimate axial strain increased by 23.2%, 45.3% and 65.5%, and the ultimate circumferential strain increased by 26.4%, 46.6% and 50.1%. The increase in fiber volume fraction had little effect on the stress of the specimen but had a significant strain on the specimen.

Both the axial and circumferential fracture strain of GFRP increased with increasing steel fiber doping, which was attributed to the confining effect of internal steel fibers. The additional constraints provided by the internal steel fiber led to improving these stress–strain behaviors. Steel fiber inhibited the development of cracks, reduced the number of isolated prominent cracks, and reduced the influence of stress concentration on GFRP. At the same time, steel fiber also improved the lateral deformation ability of core concrete, which was conducive to the restraint effect of GFRP. The fiber volume fraction was an important parameter affecting the axial compression property of the specimen, indicating that the addition of steel fiber improved the practical constraint of GFRP. The strain of the short column with fiber was significantly higher than that without fiber, but the increase in fiber content had a limited increase in strain. Considering the engineering cost and the workability of concrete, fiber content was not recommended to exceed 2%.

#### 3.2.4. Contribution of Short Concrete Columns’ Components to Compressive Strength

Table 4 lists the 7 d and 28 d compressive strength of each ratio cube test block cured under the same conditions. It can be seen from the data in the table that the strength distribution of coal gangue fiber concrete was relatively concentrated without external constraints, and the improvement of fiber on compressive strength was minimal. Taking the 28 d compressive strength of wave fiber-reinforced concrete as an example, the compressive strength of the specimens with 3%, 2% and 1% fiber was only 10.8%, 10.4% and 7.6% higher than that of the specimens without fiber. The compressive strength of a short concrete columns with four kinds of fiber content after adding the 5 mm GFRP tube was 127.43 MPa, 123.58 MPa, 123.88 MPa and 115.54 MPa, respectively, which was 176.4%, 169.1%, 176.6% and 177.7% higher than that of the specimens without constraint. From the calculation results, it could be seen that in terms of the load-carrying capacity of the short concrete column, the contribution of the GFRP tube was the largest, which was followed by the gangue concrete itself. The steel fiber contributed the least to the compressive strength of the short column, which was mainly used to increase the axial strain of the short column.

### 3.3. Test Ductility Analysis

The unconfined gangue concrete short column for the circumferential strain was about 0.005. The GFRP-confined gangue short column with three different tube thicknesses was significantly higher than the unconfined short column. The presence of a GFRP tube improved the circumferential deformation capacity of the gangue concrete short column. Still, the change in tube thickness had less effect on the circumferential deformation capacity of the short column, which was similar to the experimental results of Deng Zongcai [24]. For the axial strain, it could be seen from the figure that when the stress reached 75% of the peak stress, its growth was slow. When the stress exceeded 75% of the peak stress, the growth rate was significantly accelerated until the specimen failed. The ductility coefficient [25] was introduced to quantitatively analyze the influence of GFRP tube thickness on the axial strain of coal gangue short concrete columns.

(1) $$DI=\frac{\varepsilon_{100\%}}{\varepsilon_{y}}$$

(2) $$\varepsilon_{y}=\frac{\varepsilon_{75\%}}{0.75}$$

In the formula: *DI* is the ductility coefficient of the specimen; $\varepsilon_{100\%}$ is the corresponding axial strain when the stress reaches the peak stress, and $\varepsilon_{75\%}$ is the corresponding axial strain when the stress rises to 75% of the peak stress. The failure position of the specimen was uncertain. Occasionally, the strain gauge was pasted on the fiber tear position, and the local deformation was large, resulting in a large error. At the same time, strain gauge quality problems might also cause errors. Therefore, the specimens with large ductility coefficient errors in similar specimens were removed, and the calculation results of the remaining specimens were averaged. The results are shown in Table 5.

**Figure 6:**
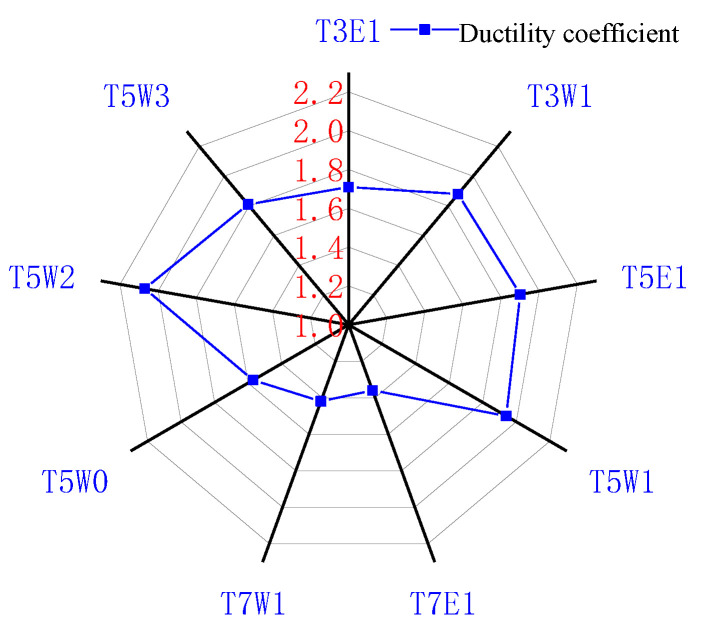
Radar distribution of ductility coefficient.

In engineering applications, in addition to improving the strength of concrete, the ductility of concrete should also be considered. T3W1, T5W1 and T7W1 were selected for comparison. The specimens with a tube thickness of 5 mm were 1.03 times and 1.37 times the specimens with a wall thickness of 3 mm and 7 mm, respectively. It can be seen that the ductility of the 3 mm and 5 mm tube thickness was better than that of the 7 mm tube thickness. T5W0, T5W1, T5W2 and T5W3 were selected for comparison. The fiber content of 1%, 2% and 3% was 1.23 times, 1.31 times and 1.15 times the fiber content of 0%, respectively. When the volume fraction of the GFRP tube was 3 mm, 5 mm and 7 mm, the ductility factor of the wave fiber was 9.6%, 2.1% and 4.4% higher than that of the end hook fiber. There was no simple linear relationship between tube thickness, fiber content, fiber shape and ductility coefficient. Some matching combinations existed to maximize the ductility coefficient of the specimen. It can be seen from Figure 6 that the ductility coefficient of the specimen with 2% wave fiber content in the specimen with 5 mm tube thickness was higher than that of the other specimens. The tube thickness and fiber volume fraction of the T5W2 specimen were the optimal combinations from the ductility point of view.

## 4. Stress Theory Analysis

### 4.1. Lateral Restraint Stress Calculation

When calculating the stress of the GFRP tube confined coal gangue short concrete column, the following formula [26] was used to calculate the lateral restraint stress:

(3) $$f_{l}=\frac{2\varepsilon_{\mathrm{frp}}E_{\mathrm{frp}}t}{d}$$

In the formula: $\varepsilon_{\mathrm{frp}}$ and $E_{\mathrm{frp}}$ are the ultimate tensile strain and elastic modulus of the GFRP tube.

### 4.2. Axial Compressive Strength Calculation of Confined Concrete

The following three models were used to calculate the axial compressive strength of confined concrete:

#### 4.2.1. The Model of Mander et al.

According to William’s failure criterion, Mander [27] obtained the formula of compressive strength of concrete under constant lateral pressure by regression analysis:

(4) $$\frac{f_{\mathrm{cc}}}{f_{\mathrm{co}}}=\left[-1.254+2.254\sqrt{1+7.94\alpha_{s}\frac{f_{l}}{f_{\mathrm{co}}}}-2\alpha_{s}\frac{f_{l}}{f_{\mathrm{co}}}\right]$$

In the formula, $f_{\mathrm{cc}}$ is the axial compressive strength of confined concrete; $f_{\mathrm{co}}$ is the axial compressive strength of unconstrained concrete; $\alpha_{s}$ is the adjustment coefficient, and $\alpha_{s}$ = 1 in this experiment.

#### 4.2.2. Deng et al. Model

Deng et al. [28] analyzed the test data of the BFRP-confined concrete cylinder in 2013 and used the least square method to perform linear regression on the test data. The strength calculation formula of the BFRP-confined concrete cylinder was as follows:

(5) $$\frac{f_{\mathrm{cc}}}{f_{\mathrm{co}}}=\left[1+3.6{\left(\frac{f_{l}}{f_{\mathrm{co}}}\right)}^{1.18}\right]$$

#### 4.2.3. Mohr–Coulomb Model (Cou)

When the core concrete was subjected to three-dimensional compression, it was in the spatial stress state of equal lateral pressure due to the lateral hoop force. The Mohr–Coulomb [29] strength formula was as follows:

(6) $$f_{\mathrm{cc}}=f_{\mathrm{co}}+\frac{1+\sin\Phi }{1-\sin\Phi }f_{l}$$

In the formula, *Φ* is the internal friction angle of concrete, and the value range is between 30° and 35°.

#### 4.2.4. Strength Calculation

The test parameters are substituted into the above three theoretical models, respectively, and the calculation results are shown in Table 6. It was seen from Table 6 that the calculation results of each model under different constraints deviated from the experimental results. For 3 mm specimens, the calculation results of the Mander model had a significant error, and the calculated value was 46.71% higher than the measured value. For 7 mm specimens, the computed values of the Deng model and Mohr–Coulomb model (Cou) showed a trend of lower than the measured values, and the Deng model and Mohr–Coulomb model (Cou) were closer to the measured values.

The deviation in the calculation results was that the above three formulas were based on ordinary concrete. Under the same strength, there were differences in the elastic modulus and failure mode between ordinary concrete and coal gangue concrete (coal gangue concrete had low elastic modulus and aggregate crushing during failure). Therefore, under compression, the contribution of core concrete to strength was weakened, and the contribution of external constraints to strength was enhanced. Therefore, it was not appropriate to use the above model to directly calculate the bearing capacity of GFRP confined coal gangue concrete short columns. We modified the existing model and converted the Deng model into Equation (7), and the modified model was denoted as Deng1.

(7) $$\frac{f_{\mathrm{cc}}}{f_{\mathrm{co}}}=A+B{\left(\frac{f_{l}}{f_{\mathrm{co}}}\right)}^{C}$$

**Figure 7:**
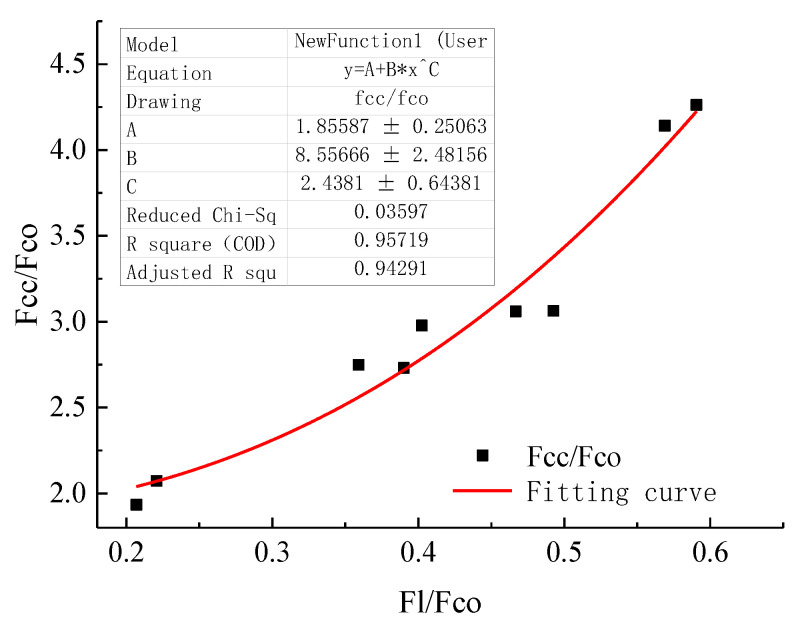
Model fitting result. “*” Represents a multiplication symbol.

Taking $\frac{f_{l}}{f_{\mathrm{co}}}$ as X and $\frac{f_{\mathrm{cc}}}{f_{\mathrm{co}}}$ as Y to make a scatter plot and fitting with Formula (7), the relevant results were obtained. As shown in Figure 7, the final A = 1.85, B = 8.5, C = 2.44, and R^2^ = 0.94, the fitting effect was good, and the modified model was recorded as Deng1. The results are shown in Table 6.

**Figure 8:**
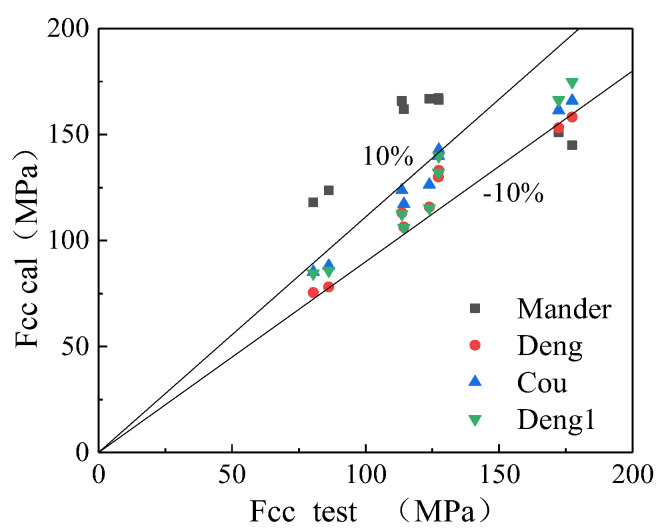
Stress scatter distribution diagram.

To show the calculation results of different models more intuitively, the comparison of the stress test values of all specimens with the stresses calculated by the Deng1 model, the Mander model, the Deng model, and the Mohr–Coulomb model (Cou) is shown in Figure 8. The modified model calculation results were more accurate.

In summary, this paper improved the bearing capacity of coal gangue concrete by external confinement GFRP, which was similar to the practice in Reference [24]. However, in this experiment, the axial compression length of confined concrete short columns was greatly improved by adding steel fiber, which improved the shortcomings of traditional coal gangue concrete with strong plasticity and poor ductility. This paper explored the influence of GFRP tube thickness, fiber type and content on the mechanical properties of coal gangue concrete. It clarified the optimal combination of GFRP tube thickness and steel fiber content on C40 coal gangue concrete for the first time, which was rare in previous articles. Finally, the classical model was used to predict the peak stress of the GFRP tube-confined coal gangue steel fiber short concrete column, and the model was modified to make it more accurate.

## 5. Conclusions and Suggestions for Future Research

This paper studied the mechanical properties of GFRP tube-confined coal gangue steel fiber short concrete composite short columns. The effects of GFRP tube thickness, steel fiber shape and fiber content on the mechanical properties of GFRP columns were analyzed. The stress–strain curves obtained from the axial compression failure of GFRP columns were analyzed, and the theoretical formula calculation was compared with the experimental data. The following conclusions were obtained:

(1) The thickness of the GFRP tube was the key factor affecting the axial compression performance of the specimen. The stress and strain of coal gangue concrete short column increased obviously with the increase in GFRP tube thickness. Compared with the unconstrained short column, the ultimate stress of the short column with tube thickness of 3, 5 and 7 mm was increased by 2.1 times, 3.0 times and 4.3 times, respectively, and the ultimate axial strain was increased by 18.8 times, 20.5 times and 21.5 times, respectively.
(2) Under a certain fiber volume fraction, the connection effect of wave fiber on concrete was more prominent. The ultimate axial strain of the wave fiber-reinforced short column of the GFRP tube thickness 3 mm specimen was 10.1% higher than that of the end hook fiber specimen. The maximum difference in ductility coefficient between the two fiber specimens was 9.6%.
(3) The steel fiber volume fraction significantly affected the axial compression behavior of the specimens. With the increase in fiber volume fraction, strain increase was more evident than stress. Compared with the specimens without fiber incorporation, the ultimate circumferential strain of the specimens with fiber volume fractions of 1%, 2%, and 3% increased by 26.4%, 46.6%, and 50.1%.
(4) In this paper, by comparing the typical axial stress model of the existing literature with the experimental data, it was found that they all had different degrees of error. A stress model suitable for GFRP tube-confined steel fiber-reinforced coal gangue concrete was proposed by comparing the three stress models. The modified Deng model was in good agreement with the experimental results.

Future Research: This study optimized and upgraded the existing experiments and theories, but there were still some limitations. For example, the location of the strain gauge determines that they could only detect the strain of the GFRP tube. When the GFRP tube slipped with the core concrete, the monitoring data could not truly reflect the state of the concrete. In addition, due to time constraints, only one form of GFRP fiber tube was used in the test, and no other types of fiber-reinforced polymers were tested. The type of fiber tube will be further explored in future tests.

## Figures and Tables

**Table 1:** Details of specimens.

Serial Number	Tube Thickness (mm)	Fiber Type	Fiber Content (%)	Specimen Height (mm)	Specimen Diameter (mm)
T3E1	3	End-hooked fiber	1	300	150
T3W1	3	Wave fiber	1	300	150
T5E1	5	End-hooked fiber	1	300	150
T5W1	5	Wave fiber	1	300	150
T7E1	7	End-hooked fiber	1	300	150
T7W1	7	Wave fiber	1	300	150
T5W0	5	Wave fiber	0	300	150
T5W2	5	Wave fiber	2	300	150
T5W3	5	Wave fiber	3	300	150
T0W1	0	Wave fiber	1	300	150

‘T’ represents the thickness of the GFRP tube, ‘E’ and ‘W’ represent the end hook fiber and wave fiber, respectively, and ‘T3E1’ represents the thickness of the GFRP tube 3 mm end hook fiber content of 1%.

**Table 2:** Mix proportion of coal gangue concrete.

Material	Cement	River Sand	Coal Gangue	Water	Water-Reducing Admixture
Proportion	15.60%	35.70%	41.28%	7.01%	0.36%

**Table 3:** Properties of FRP.

Type of FRP	Tensile Strength (MPa)	Elastic Modulus (GPa)	Bending Strength (MPa)
CFRP	3165.4	224.5	57
GFRP	430	22	138

5 mm thick GFRP tube as an example.

**Table 4:** Compressive strength of concrete cube test block under the same condition curing.

Specimen Number	7 d Strength/MPa	28 d Strength/MPa	Specimen Number	7 d Strength/MPa	28 d Strength/MPa
T3E1	33.21	42.94	T7W1	34.57	45.16
T3W1	35.14	44.46	T5W0	31.52	41.61
T5E1	33.54	43.13	T5W2	35.72	45.92
T5W1	34.75	44.12	T5W3	36.23	46.11
T7E1	33.18	42.82	T0W1	35.31	45.41

**Table 5:** Ductility coefficient of specimens and comparison.

Serial Number	*ε_y_*	*ε*_100%_	*DI*
T3E1	0.01040	0.01777	1.71
T3W1	0.01041	0.01956	1.88
T5E1	0.01207	0.02298	1.90
T5W1	0.01361	0.02647	1.94
T7E1	0.02877	0.03912	1.36
T7W1	0.02832	0.04014	1.42
T5W0	0.01371	0.02148	1.57
T5W2	0.01505	0.03122	2.07
T5W3	0.01959	0.03554	1.81

**Table 6:** Model calculation results and deviations.

Serial Number	Stress Measured Value (MPa)	Mander		Cou		Deng		Deng1	
		Calculated Value (MPa)	Increasing Range (%)	Calculated Value (MPa)	Increasing Range (%)	Calculated Value (MPa)	Increasing Range (%)	Calculated Value (MPa)	Increasing Range (%)
T3E1	80.43	118.01	46.71%	85.17	5.89%	75.43	−6.22%	84.53	5.09%
T3W1	86.20	123.57	43.37%	88.10	2.21%	78.13	−9.36%	85.83	−0.42%
T5E1	113.59	165.78	45.95%	123.72	8.92%	113.08	−0.45%	112.51	−0.95%
T5W1	123.89	166.81	34.64%	126.33	1.98%	115.77	−6.55%	115.33	−6.91%
T7E1	172.28	151.10	−12.30%	161.40	−6.32%	153.21	−11.07%	166.28	−3.48%
T7W1	177.33	144.98	−18.25%	165.95	−6.42%	158.23	−10.77%	174.79	−1.44%
T5W0	114.31	161.95	41.68%	117.22	2.55%	106.45	−6.88%	106.03	−7.25%
T5W2	127.28	167.28	31.42%	139.88	9.90%	129.95	2.10%	132.06	3.75%
T5W3	127.43	166.30	30.50%	142.84	12.09%	133.10	4.45%	139.77	9.68%

## Data Availability

All the data will be available to the readers.
